# Preserved Intention Maintenance and Impaired Execution of Prospective Memory Responses in Schizophrenia: Evidence from an Event-based Prospective Memory Study

**DOI:** 10.3389/fpsyg.2016.00593

**Published:** 2016-04-28

**Authors:** Gyula Demeter, István Szendi, Nóra Domján, Marianna Juhász, Nóra Greminger, Ágnes Szőllősi, Mihály Racsmány

**Affiliations:** ^1^Frontostriatal System Research Group, Hungarian Academy of SciencesBudapest, Hungary; ^2^Department of Cognitive Science, Budapest University of Technology and EconomicsBudapest, Hungary; ^3^Department of Psychiatry, University of SzegedSzeged, Hungary

**Keywords:** schizophrenia, prospective memory, executive functions, intention maintenance, negative symptoms

## Abstract

Executive system dysfunction and impaired prospective memory (PM) are widely documented in schizophrenia. However, it is not yet clarified which components of PM function are impaired in this disorder. Two plausible target components are the maintenance of delayed intentions and the execution of PM responses. Furthermore, it is debated whether the impaired performance on frequently used executive tasks is associated with deficit in PM functions. The aim of our study was twofold. First, we aimed to investigate the specific processes involved in event-based PM function, mainly focusing on difference between maintenance of intention and execution of PM responses. Second, we aimed to unfold the possible connections between executive functions, clinical symptoms, and PM performance. An event-based PM paradigm was applied with three main conditions: baseline (with no expectation of PM stimuli, and without PM stimuli), expectation condition (participants were told that PM stimuli might occur, though none actually did), and execution condition (participants were told that PM stimuli might occur, and PM stimuli did occur). This procedure allowed us to separately investigate performances associated with intention maintenance and execution of PM responses. We assessed working memory and set-shifting executive functions by memory span tasks and by the Wisconsin Card Sorting Test (WCST), respectively. Twenty patients diagnosed with schizophrenia and 20 healthy control subjects (matched according to age and education) took part in the study. It was hypothesized that patients would manifest different levels of performance in the expectation and execution conditions of the PM task. Our results confirmed that the difference between baseline performance and performance in the execution condition (execution cost) was significantly larger for participants diagnosed with schizophrenia in comparison with matched healthy control group. However, this difference was not observed in the expectation condition. The PM performance in the execution condition was correlated with impaired executive functions in schizophrenia. Specifically, the size of execution cost positively correlated with percent of perseverative errors committed on WCST by the patient group. Our results suggest that maintenance of delayed intentions is unimpaired in schizophrenia, whereas the impairment in execution of PM responses is associated with set-shifting deficit.

## Introduction

Prospective memory (PM) refers to functions of encoding, storage, and retrieval of delayed intentions (Ellis, 1996; Graf and Uttl, 2001) involving consecutive steps of information processing, such as formation, retention, initiation, and execution of intentional acts (Kliegel et al., 2002). Three main categories of PM tasks are distinguished in the literature, called *event-based* (remembering to perform an intention when a cue appears), *time-based* (remembering to perform an intention at a specific time or after a period of time), and *activity-based* (remembering to perform an intention upon the completion of an activity) PM tasks (Einstein and McDaniel, 1996). In a typical experimental setting, PM is typically assessed by a dual-task paradigm where the PM task is embodied in an ongoing task (Kliegel et al., 2001; Guynn, 2003, 2008; Marsh et al., 2005).

It is generally assumed that processes of intention formation, initiation and execution of PM responses involve specific executive components associated mainly with frontal networks (Okuda et al., 1998; Burgess et al., 2001, 2003; Simons et al., 2006; Zöllig et al., 2007; Kliegel et al., 2008), whereas intention retention which requires long-term memory processes is supported mainly by hippocampal structures (Cohen and O’Reilly, 1996; Guynn et al., 2001; Kliegel et al., 2008). Dominant theories of PM consider differently the role of executive control processes in PM tasks. There are theories, such as the *preparatory attentional and memory processes model* (Smith, 2003; Smith and Bayen, 2004) and the *two-component theory* of PM (Guynn, 2008), which assume that the involvement of the executive system or controlled attention is critical in carrying out adequate PM responses, whereas the *multiprocess model* (McDaniel and Einstein, 2000; McDaniel et al., 2004) proposes that automatic processes can trigger PM responses if the PM cue and the response are strongly associated.

It is widely documented that the executive control system is impaired in various patient populations; however, its relation to PM performance is still under debate. Patients diagnosed with schizophrenia along with a general impairment in tasks measuring executive processes, frequently manifest difficulties in PM tasks during their everyday activity, such as remembering the medical appointments and taking their medication at the appropriate time (Shum et al., 2001; Ungvari et al., 2008).

Based on earlier relevant findings there has been consistent evidence that PM impairment is present also at illness onset (Zhou et al., 2012, Zhuo et al., 2013; Lui et al., 2014) and at the chronic stage (Elvevåg et al., 2003; Shum et al., 2004; Henry et al., 2007; Woods et al., 2007; Altgassen et al., 2008; Chan et al., 2008; Twamley et al., 2008; Wang et al., 2008). The results regarding the findings in the event- and time-based PM tasks most frequently used are inconsistent. There are results showing that patients manifest similar impairments in the two tasks (e.g., Henry et al., 2007; Woods et al., 2007; Lui et al., 2011), whereas others found greater impairment on time-based compared to on event-based PM tasks (e.g. Shum et al., 2004; Wang et al., 2008; Cheung et al., 2015).

In a meta-analytic review based on 11 studies Wang et al. (2009) concluded that schizophrenic patients showed greater deficit on time-based tasks compared to performance on event-based tasks. They also found that PM performance negatively correlated with medication dosage, negative symptoms, general psychopathology, duration of illness, age, education, and IQ (for details see Wang et al., 2009). A more recent systematic review based on 22 studies supports the previous findings showing serious PM impairment in schizophrenia compared to healthy controls, along with the lack of awareness of patients about their difficulties. However, according to this study, PM performance did not associate with medication dosage or with chronicity of illness (for details see Ordemann et al., 2014). A series of studies found impaired PM performance in comparison with IQ matched control group, without any group difference on executive and memory tasks, suggesting that PM is a primary rather than a secondary deficit (Henry et al., 2007; Chan et al., 2008; Wang et al., 2008). Woods et al. (2007) suggested that in schizophrenia the PM deficit is due to the impaired cue detection and self-initiated intention retrieval. Taken together, these studies indicate that PM impairment is present in patients with schizophrenia; however, it is still unclear whether PM deficit is strongly associated to a general executive impairment, and which processes are impaired in PM functioning. Interestingly, despite the plethora of studies focusing on PM in schizophrenia, to our best knowledge, there is no published study in which the applied paradigms made methodologically possible the distinct analysis of maintenance and execution processes in PM. Here, we suggest that investigating both intention maintenance and execution of PM responses is crucial for understanding the nature of PM deficit in schizophrenia, as these functions are mediated by different executive processes and recruiting distinct neuroanatomical networks.

From the neuroimaging literature it is known that in schizophrenia there is a functional connectivity impairment, mainly affecting prefrontal–basal ganglia circuits and prefrontal–temporal connections (Friston and Frith, 1995; Andreasen et al., 1999; Stephan et al., 2006). A vast amount of data also showed decreased activity in various prefrontal areas (Berman and Weinberger, 1991; Andreasen et al., 1992; Volz et al., 1997; Perlstein et al., 2001; Meyer-Lindenberg et al., 2002; Ranganath et al., 2008). These critical cortical areas seem to play crucial roles in prospective remembering as well, as it was demonstrated by a series of neuroimaging studies with healthy participants showing that rostral prefrontal cortex (BA10) is involved in PM (e.g., Burgess et al., 2001; Simons et al., 2006; Benoit et al., 2012). In a positron emission tomography (PET) study Burgess et al. (2001) demonstrated that different cortical areas are involved in maintaining and realizing intentions. Furthermore, there is evidence that PM is more sensitive to executive deficits than to retrospective memory impairments (Kopp and Thöne-Otto, 2003; Kliegel et al., 2004b), supporting the idea that PM strongly relies on executive control networks involving prefrontal cortex (McDaniel et al., 1999; Brunfaut et al., 2000). Others suggest that not PM function as such, but only specific subprocesses of PM – such as planning of an intention or the initiation and execution of PM responses requiring executive control – and rely on the prefrontal cortex, whereas other PM subcomponents such as the retrieval of the intention content, relying on medial temporal lobe networks (Cohen and O’Reilly, 1996; Guynn et al., 2001). Based on all these results we can assume that different cortical networks are responsible for intention maintenance and intention execution impairments. If there is a dysfunction in the networks of frontal pole (Br. 10), the right lateral prefrontal cortex and inferior parietal cortex both intention maintenance and execution could be impaired, whereas a functional deficit of thalamus and dorsolateral prefrontal cortex could produce disorder of execution PM responses.

The aim of our study using a computer-based dual-task paradigm was twofold. First we wanted to acquire further evidence about the deficit of event-based PM in schizophrenia. More specifically, our first research goal was to study the possible differences between maintenance of intention and its behavioral realization in this disorder. Second, we aimed to unveil possible connections between executive functions, clinical symptoms and PM performance in schizophrenia. To fulfill this aim we choose a specific event-based PM task applied earlier in the above mentioned imaging study (Burgess et al., 2001) and in a study with psychiatry population, patients diagnosed with obsessive–compulsive disorder (OCD; Racsmány et al., 2011). Earlier we gained evidence that administering this task for patient population would not produce a difference in hit rates between patients with executive disorders and matched healthy control groups (Racsmány et al., 2011), suggesting that any possible difference in terms of reaction times would not be the consequence of performance/time trade-offs.

According to Miyake et al. (2000), traditional neuropsy chological executive tasks load on three main central executive components: inhibition, modality specific updating–monitoring, and shifting. Inhibition refers to one’s ability to deliberately inhibit dominant, automatic, or prepotent responses when necessary. Updating and monitoring refer to the refreshing and actively manipulating the content of working memory, rather than passively store information. The shifting component of the executive system is responsible for the coordination of the change between task-relevant and task-irrelevant sets.

We used short-term memory span tasks as measure of working memory function (Baddeley and Hitch, 1974; Baddeley, 1986, 2001) conceptualized also as an updating component of the executive system (Miyake et al., 2000), and the Wisconsin Card Sorting Task (WCST) to measure a specific component of executive system, the shifting component, usually associated to impairment on fluency and dual tasks.

Here, based on earlier results, we hypothesized that patients will manifest difficulties in executive shifting task and also in the execution part of the event-based PM task requiring executive control resources.

## Materials and Methods

### Participants

Twenty patients diagnosed with schizophrenia were enrolled from the outpatient care of the Department of Psychiatry, University of Szeged, Hungary (mean age = 38.55 years, *SD* = 10.24; mean education = 11.3 years, *SD* = 2.39). They were all in stable interepisodic states taking antipsychotic medication. All substances [seven kinds of second generation antipsychotics (SGA); one depot first generation antipsychotic (FGA); four SGA+SGA and one SGA+FGA combination] were prescribed according to their medication protocols, the mean dose in chlorpromazine equivalence (CPZ) was 375.36 mg/die (*SD* = 385.05; min = 50, max = 1620). Patients were diagnosed (American Psychiatric Association, 2000) and evaluated clinically by an expert psychiatrist (IS) using the Positive and Negative Syndrome Scale (PANSS; Kay et al., 1987). The exclusion criteria were related to possible organic brain dysfunctions (a lifetime history of neurological illness, any medical illness known to affect brain structure, head injury with loss of consciousness for more than 10 min) that could significantly constrain neurocognitive performance. We succeeded in enrolling patients with both the more favorable and unfavorable courses.

The healthy control group was matched according to age and education (*n* = 20, mean age = 35.75 years, *SD* = 10.24; mean education = 12.15 years, *SD* = 2.16). Control participants with a personal history of psychiatric disorder or a family history of psychotic and affective spectrum disorders, history of neurological illness and substance abuse were excluded. They were screened for Axis I disorders by the means of the Mini-International Neuropsychiatric Interview (Balázs et al., 1998; Sheehan et al., 1998).

All participants gave written informed consent and the protocol was approved by the Human Investigation Review Board, University of Szeged, Albert Szent-Györgyi Medical and Pharmaceutical Centre, and it was carried out in accordance with the latest version of the Declaration of Helsinki (see **Table 1** for participants’ characteristics).

**Table 1:** Sample demographics.

Characteristics	SCH (*n* = 20)		HC (*n* = 20)	
	Mean	*SD*	Mean	*SD*
Age (years)	38.55	10.24	35.75	10.24
Education (years)	11.3	2.39	12.15	2.16
Sex (M/F)	11/9		9/11	
Full scale IQ WAIS-H	99	15.96	110.65	11.31
PANSS positive symptoms	11.25	4.62		
PANSS negative symptoms	14.9	6.29		
PANSS total scores	55	19.27		

*SCH, patients with schizophrenia; HC, healthy controls; M, male; F, female; WAIS-H, Wechsler Adult Intelligence Scale – Hungarian version, PANSS, Positive and Negative Syndrome Scale.*

### Experimental Design and Procedure

At the beginning of the experimental session all patients were screened with a neuropsychological battery, focusing on verbal and visuo-spatial working memory (Digit Span Forward and Backward Tasks and Corsi Block Design Task, respectively) and executive functions (WCST).

An event-based PM task was administered to each participant under three conditions: (i) a *baseline condition* in which there was no expectation that PM stimuli would occur, and no PM stimuli occurred; (ii) an *expectation condition* in which participants were told that PM stimuli might occur, though none actually did; and (iii) an *execution condition* in which participants were told that PM stimuli might occur, and PM stimuli did occur. This procedure allowed us to distinguish the performances associated with intention maintenance and its realization (Burgess et al., 2001; Racsmány et al., 2011).

Sixty stimuli were presented in the baseline and expectation conditions. The execution condition contained PM stimuli that were pseudorandomly distributed, amounting to 25% of the stimuli. In each condition, the first six stimuli were considered practice items and were not included in the analysis. We administered the baseline condition first and we counterbalanced the expectation and execution conditions for all participants. Stimuli presentation strictly adhered to the Burgess et al. (2001) procedure and was subject-paced (i.e., the onset of the next stimulus was cued by the subject’s response and the stimuli remained visible until that response occurred). A 2000 ms blank white screen interval with a fixation cross was inserted between presentations.

**FIGURE 1:**
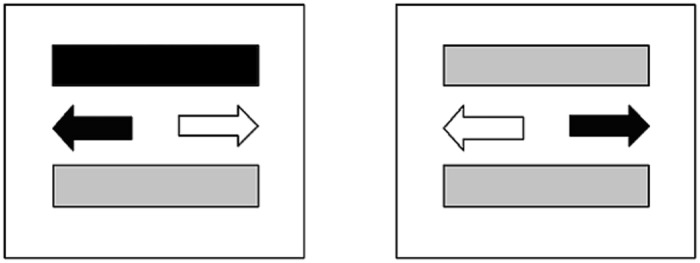
**The event-based PM task.** Description of the tasks: (i) Ongoing task: Press the key (left or right) in the direction of black arrow; (ii) PM task: If the two color bars are the same color, press the up-arrow key.

In each trial, two arrows were presented on the display. One arrow was always black, and its position varied pseudorandomly. In both the baseline and expectation conditions, stimuli included 30 items in which the black arrow pointed to the left and an additional 30 items in which it pointed to the right. The ratio in the execution condition was 40/40. Two color bars also appeared on the screen and were located at equal distances above and below the arrows. The color of the horizontal bar was red, blue, green, yellow, or orange (**Figure 1**).

Written instructions were read to the participants immediately before each experimental block was administered. Participants were asked to press the key with their forefinger if the black arrow was on the left of a fixation point and with their third finger if it was on the right. In the expectation and execution conditions, participants were told to respond with their middle finger if the two color bars above and below the fixation point were the same color on any trial, this instruction served as a PM task.

## Results

Similarly to previous studies of Burgess et al. (2001) and Racsmány et al. (2011), errors for non-PM and PM stimuli were rare. Hit rate was above 90% in the PM task, and above 97 % in the ongoing tasks in all the three experimental conditions in both groups.

**FIGURE 2:**
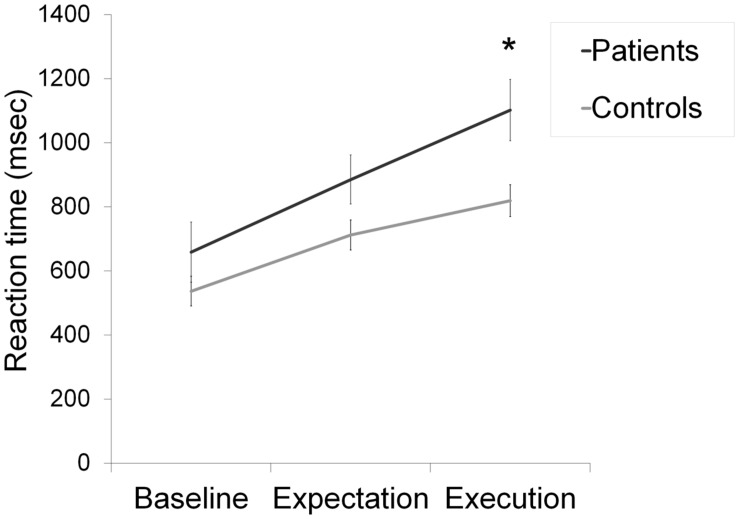
**Mean reaction times by condition for the ongoing task.** Error bars show standard error of the mean, ^∗^*p* < 0.05.

**FIGURE 3:**
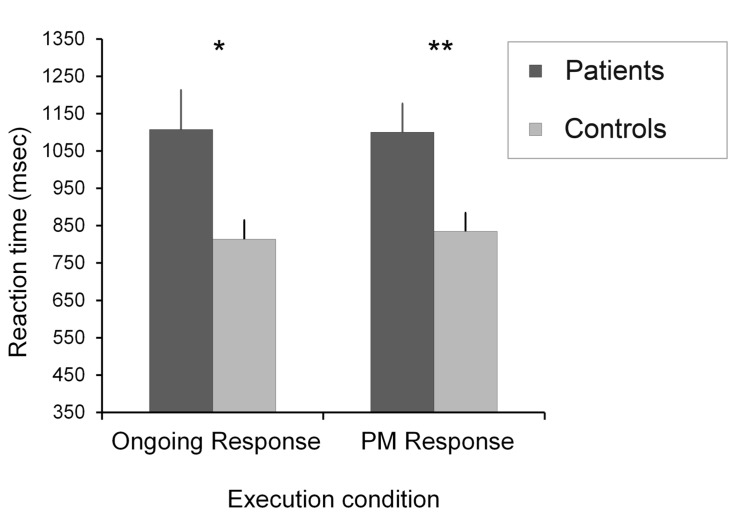
**Mean reaction times for the ongoing and PM tasks in the execution condition.** Error bars show standard error of the mean, ^∗^*p* < 0.05; ^∗∗^*p* < 0.01.

Mean reaction times (RTs) for the ongoing task were analyzed in a group (patients and healthy control) × condition (baseline, expectation, and execution) repeated measures ANOVA. Analysis of RTs was based on errorless trials. The group (patients and healthy control) × Condition (baseline, expectation, and execution) repeated measures ANOVA for the participants’ mean RTs in the ongoing task showed a significant main effect of condition [*F*(2,38) = 147.66, *p* < 0.001, and $\eta_{p}^{2}=0.79$] and a significant effect of group [*F*(2,38) = 4.07, *p* = 0.05, and $\eta_{p}^{2}=0.09$]. There was a significant group × condition interaction [*F*(2,38) = 8.35, *p* < 0.01, and $\eta_{p}^{2}=0.18$]. We found a significant difference between the two groups [*t*(39) = 2.52, *p* < 0.05, and *r* = 0.37] only in the ongoing task of the execution condition. There was no significant difference in the baseline condition [*t*(39) = 1.16, *p* > 0.05, and *r* = 0.18], and in the expectation condition [*t*(39) = 1.93, *p* > 0.05, and *r* = 0.29] (**Figure 2**). Comparison of the patient’s and the healthy control group’s RTs in the PM task of the execution condition [*t*(39) = 2.91, *p* < 0.01, and *r* = 0.42] revealed significant differences (**Figure 3**). In sum, the patient group performed significantly slower in the ongoing and PM tasks of the execution condition.

**FIGURE 4:**
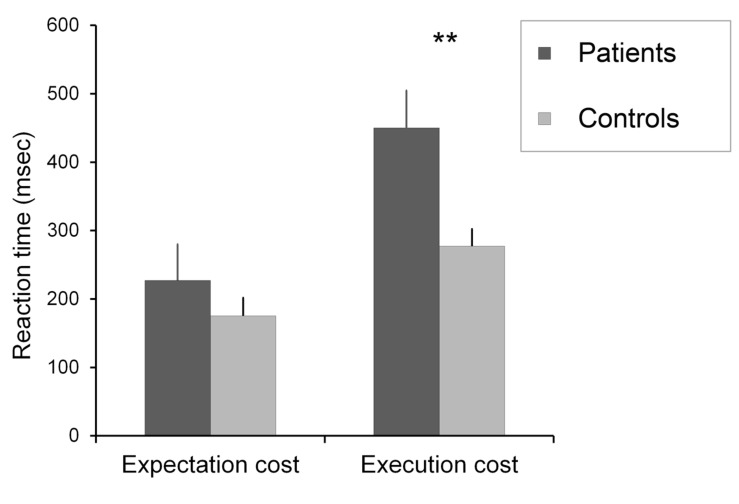
**The expectation and execution costs in the two groups.** Error bars show standard error of the mean, ^∗∗^*p* < 0.01.

To further analyze our data, following Burgess et al. (2001) a “cost of PM instruction” was calculated for both the expectation condition (mean ongoing task RT in the expectation condition – mean ongoing task RT in the baseline condition) and the execution condition (mean ongoing task RT in the execution condition – mean ongoing task RT in the baseline condition). Comparison of expectation costs revealed no significant difference [*t*(39) = 0.88, *p* > 0.05, and *r* = 0.14], whereas the same comparison yielded a significant difference for execution costs [*t*(39) = 2.89, *p* < 0.01, and *r* = 0.42] between the two groups, the patient group slowed down significantly (**Figure 4**).

Results on the neuropsychological tasks for the two groups are summarized in **Table 2**.

**Table 2:** Neuropsychological assessment.

Tasks	SCH (*n* = 20)		HC (*n* = 20)		*t*	*p*
	Mean	*SD*	Mean	*SD*		
DSF	6.2	1.47	5.9	1.41	0.66	n.s.
DSB	4.15	0.81	4.6	1.09	−1.47	n.s.
CBDT	5.3	1.26	5.35	0.81	−0.15	n.s.
WCST CR	42.95	28.91	70.89	17.19	−3.62	<0.001
WCST CC	3.1	2.38	5.2	1.75	−3.11	<0.01
WCST PE	26.95	21.49	12.95	7.49	2.68	<0.05

*SCH, patients with schizophrenia; HC, healthy controls; DSF, Digit Span Forward; DSB, Digit Span Backward; CBDT, Corsi Block Design Task – forward; WCST CR, Wisconsin Card Sorting Test, percent conceptual level responses; WCST CC, Wisconsin Card Sorting Test, categories completed; WCST PE, Wisconsin Card Sorting Test, percent preservative errors; n.s., not significant.*

Two sets of Pearson’s correlational analysis (two-tailed) were conducted. The first involved only the data from patient group and examined the relationship between clinical symptoms and neuropsychological tasks and main PM task indices (for results see **Table 3**). The second analysis included both groups and focused on the possible relationships between WCST relevant scores and PM task executive scores (for results see **Table 4**). Bonferroni corrections were applied to each correlational matrix for each experimental group.

**Table 3:** Correlations among clinical symptoms, cognitive functions, and event-based PM task scores in patients with schizophrenia.

	SCH (*n* = 20)	
	PANSS Positive	PANSS Negative
**Working memory**		
DSF	0.04	−0.16
DSB	−0.04	−0.31
CBDT	0.07	−0.04
**Set-shifting**		
WCST CC	−0.19	−0.48^∗^
WCST PE%	0.09	0.33
**PM**		
Baseline condition ongoing task RT	0.19	0.55^∗^
Expectation condition ongoing task RT	0.29	0.50^∗^
Execution condition ongoing task RT	0.39	**0.70**^∗∗^
Execution condition PM task RT	0.38	0.60^∗∗^
Expectation cost	0.09	−0.25
Execution cost	0.43	0.40

SCH, patients with schizophrenia; PANSS, Positive and Negative Syndrome Scale; DSF, Digit Span Forward; DSB, Digit Span Backward; CBDT, Corsi Block Design Task – forward; WCST CC, Wisconsin Card Sorting Test, categories completed; WCST PE%, Wisconsin Card Sorting Test, percent perseverative errors; PM, prospective memory; RT, reaction time; Expectation Cost, mean ongoing task reaction time in the expectation condition – mean ongoing task reaction time in the baseline condition; Execution cost, mean ongoing task reaction time in the execution condition – mean ongoing task reaction time in the baseline condition; *p < 0.05 (two tailed); **p < 0.01 (two-tailed). The statistics that remained significant after Bonferroni correction are in bold (adjusted cut-off oxp-value of 0.002).

**Table 4:** Correlations among PM task executive condition indices and WCST main scores in patients with schizophrenia and healthy controls.

	SCH (*n* = 20)		HC (*n* = 20)	
	WCST	WCST	WCST	WCST
	CC	PE	CC	PE
Execution condition Ongoing Task RT	**-0.64**^∗∗^	0.44	−0.09	0.49^∗^
Execution condition PM Task RT	−0.55^∗^	0.35	−0.03	0.38
Execution cost	−0.42	**0.60**^∗∗^	−0.07	−0.01

*SCH, patients with schizophrenia; HC, healthy controls; WCST CC, Wisconsin Card Sorting Test, categories completed; WCST PE%, Wisconsin Card Sorting Test, percent perseverative errors; PM Task, prospective memory task; RT, reaction time; Execution cost, mean ongoing task reaction time in the execution condition – mean ongoing task reaction time in the baseline condition; ^∗^p < 0.05 (two-tailed); ^∗∗^p < 0.01 (two-tailed). The statistics that remained significant after Bonferroni correction are in bold (adjusted cut-off oxp-value of 0.008).*

To investigate the possible effects of medication on PM performance we analyzed the relationship between medication dosage and PM indices. We found no significant correlation between CPZ rates and RT data. Specifically, Pearson’s correlations between CPZ rates and Mean RTs for ongoing task in the baseline condition were *r* = 0.009, n.s.; in the expectation condition *r* = 0.158, n.s.; and in the execution condition *r* = 0.095, n.s. Cost of PM instruction did not correlate with CPZ rates either in the expectation or in the execution condition (*r* = 0.207, n.s.; *r* = 0.146, n.s., respectively). The mean RTs of the PM task in the execution condition also did not correlate with CPZ rates (*r* = 0.170, n.s.).

## Discussion

The present study examined PM functions in patients diagnosed with schizophrenia using a computerized event-based PM paradigm with three conditions: single ongoing task; ongoing task with expectation of PM stimuli, but without PM stimuli; and ongoing task interspersed with PM stimuli. This procedure lent itself to separately analyze two major phases of PM: intention maintenance and intention realization. There are two main findings. First, patients diagnosed with schizophrenia in comparison with healthy control group showed altered PM performance in the execution condition and not in the expectation condition. In other words, patients with schizophrenia showed significantly slower performance in executing PM responses, whereas their performance was in the normal range in a task associated with maintenance of PM intentions. The patient group responded significantly slower for the presence of the PM cue, and showed longer reaction times both for the PM stimuli and for the ongoing task stimuli in the execution condition. Our second finding revealed by further analysis that the PM cost in execution condition correlated with executive set-shifting deficit measured by WCST. Our results are consistent with previous findings indicating that executive functions are impaired in schizophrenia (Abbruzzese et al., 1995, 1997; Heinrichs and Zakzanis, 1998; Velligan and Bow-Thomas, 1999; Matza et al., 2006; Snitz et al., 2006; Racsmány et al., 2008; Raffard and Bayard, 2012; Green and Harvey, 2014) and the size of executive impairment associated with the level of performance in event-based PM tasks (for reviews see Wang et al., 2009; Ordemann et al., 2014).

The patients involved in our study produced intact working memory performance, whereas their executive shifting performance measured by the WCST was impaired. Patients committed significantly more perseverative errors, produced fewer conceptual level responses and achieved fewer categories than the matched healthy controls. The higher number of perseverative errors is a good index of executive set-shifting deficit.

Based on our results, we suggest that deficit of the executive shifting function in schizophrenia is strongly related to their specific PM dysfunction. This idea is underlined by the present result showing that preservative error rate in WCST positively and strongly correlated with the increased execution cost on the PM task (*r* = 0.60 and *p* < 0.01).

There is a debate in the literature whether PM impairment is a primary deficit in schizophrenia or whether PM impairment is only a consequence of other cognitive deficits (secondary deficit). According to Henry et al. (2007), PM impairment in schizophrenia could not be explained by other cognitive difficulties. In contrary, Kondel (2002) demonstrated that schizophrenic patients with intact executive functions were better on PM tasks than those who had executive deficits. Other more recent PM studies also support the idea that executive deficit contributes to PM dysfunction in schizophrenia (see Shum et al., 2004; Twamley et al., 2008; Lui et al., 2011). Based on our results, we suggest that the executive attentional shifting component of the executive system plays an important role in the execution of PM responses.

Inconsistent results in the literature might be the consequence of differences in PM tasks applied in earlier studies, showing significant differences in task difficulty. A large part of earlier studies applied *dual-task paradigms* (e.g., Elvevåg et al., 2003; Shum et al., 2004; Woods et al., 2007; Altgassen et al., 2008; Chan et al., 2008; Twamley et al., 2008; Ungvari et al., 2008; Wang et al., 2008; Zhuo et al., 2013; Lui et al., 2014), whereas other studies used so-called *ecologically valid PM paradigms*, for instance the Virtual Week or the Cambridge PM Task (Henry et al., 2007; Zhuo et al., 2012). We think that task demands in each study, including the difficulty of the ongoing task, the frequency and saliency of PM cues, the time between intention formation and execution may affect subject’s performance on these tasks as well.

A couple of PM studies (Woods et al., 2007; Twamley et al., 2008) with patients diagnosed with schizophrenia found a positive relationship between negative symptoms and PM performance. In accordance with these results we also found a strong positive correlation between negative symptoms and ongoing task scores in the execution condition (*r* = 0.70 and *p* < 0.001). Importantly, a meta-analyses based on the results of 15 published studies showed that negative symptoms are associated to impaired executive performance (Nieuwenstein et al., 2001). In line with this suggestion, we found correlation between negative symptoms and WCST scores, however, this result did not survive Bonferroni correction, suggesting a lack of statistical power in our study. We found no significant correlation between CPZ rates and PM indices indicating that event-based PM performance in our patient group is not a side-effect of medication. This is in line with Zhuo et al. (2012) findings with drug-naïve patients, who demonstrated PM deficit when compared to healthy controls.

Studies with different patient populations also support the possible link between executive functions and PM. In our previous study using the same experimental paradigm with patients suffering from OCD we found similar results, with the exception that OCD patients slowed down significantly also in the expectation condition of the PM task (for details see Racsmány et al., 2011). The executive deficit in OCD is well documented (for reviews see Kuelz et al., 2004; Olley et al., 2007; Abramovitch and Cooperman, 2015). OCD patients following PM instructions produced a type of over-activation in monitoring for PM cues and we have argued that impaired inhibition component of the executive system could be responsible for this “hyperactivity”. It is possible that OCD patients are unable to inactivate successfully realized intentions and due to inhibition deficit the previous task remains on their “to execute list” and this contributes then to the observed obsessive–compulsive behavior. Unlike OCD subjects schizophrenic patients did not produce longer reaction time scores in the expectation condition compared to the healthy controls, however, when the PM cues were really present they responded significantly slower compared to the healthy controls, probably due to set shifting difficulties from the ongoing to the PM task. This result can be interpreted also in the conceptual framework of the PAM theory (Smith, 2003; Smith and Bayen, 2004), patients in those PM tasks which resolution requires increased attentional control will manifest impaired performance due to impaired executive functions.

Our results also could be linked to the findings with brain injured patients who showed executive function deficits along with impaired PM performance (Daum and Mayes, 2000; Maujean et al., 2003; Kliegel et al., 2004a). However, we also need to note that a considerable number of studies with traumatic brain injured patients found deficit in different PM task without detected executive function deficit (Bisiacchi, 1996; Burgess, 2000; Fortin et al., 2002, 2003).

The main behavioral findings of this study may have some consequence in light of neuroimaging studies of PM. A series of studies found evidence that the rostral prefrontal cortex (BA10) is involved in PM (Burgess et al., 2001, 2003; Simons et al., 2006). It was demonstrated by Burgess et al. (2001) in a positron emission tomography (PET) study applying the same event-based PM task than in the present study, that different cortical areas are involved in the maintenance and the realization of intentions. Burgess et al. (2001) found increased regional cerebral blood flow (rCBF) in the frontal pole bilaterally, in the right lateral prefrontal, inferior parietal cortex and the precuneus, and decreased rCBF in the insula in the left hemisphere when healthy participants maintained and realized an intention. In contrast, Burgess et al. (2001) found increased rCBF in the right thalamus and decreased rCBF in the right dorsolateral prefrontal cortex (DLPFC) when subjects executed versus maintained an intention. Regarding a range of neuroimaging findings showing decreased activity of the DLPFC recruited by executive tasks in schizophrenia (Volz et al., 1997; Perlstein et al., 2001; Meyer-Lindenberg et al., 2002; Manoach, 2003), a plausible interpretation of our findings is that impairment of DLPFC-related networks is responsible for impaired performance on WCST and event-based PM task. Further neuroimaging studies are needed to clarify these assumptions.

The limitation of our study must be considered in interpreting the conclusions. Considering the heterogeneity of schizophrenia the relatively low number of participants could be a major limitation of the present results. The use of additional executive tasks and long-term memory probes could be useful as well to help clarifying the possible relationship between executive functions and PM.

In summary based on these findings it is suggested that PM deficit in schizophrenia is mainly related to the execution of PM responses and not to the maintenance of prospective intentions (see Kliegel et al., 2011). At the same time our study identified executive attentional set-shifting functions measured by WCST, as part of the executive control system, as the core cognitive function associated with PM impairment, what should be addressed during cognitive-behavioral training protocols in order to improve PM function in patients living with schizophrenia contributing to their everyday wellbeing.

## Author Contributions

Authors MR and GD designed the study and wrote the protocol. GD undertook the statistical analysis and wrote the first draft of the manuscript. IS organized the study and commented the first and final draft of the manuscript. MR wrote the final draft of the manuscript. ND, MJ, NG, and ÁS assisted to data acquisition and commented the first draft of the manuscript. All authors contributed and have approved the final manuscript.

## Conflict of Interest Statement

The authors declare that the research was conducted in the absence of any commercial or financial relationships that could be construed as a potential conflict of interest.

## References

[B1] Abbruzzese M. Bellodi L. Ferri S. Scarone S. (1995). Frontal lobe dysfunction in schizophrenia and obsessive-compulsive disorder: a neuropsychological study. *Brain Cogn.* 27 202–212. 10.1006/brcg.1995.1017 7772333

[B2] Abbruzzese M. Ferri S. Scarone S. (1997). The selective breakdown of frontal functions in patients with obsessive-compulsive disorder and in patients with schizophrenia: a double dissociation experimental finding. *Neuropsychologia* 35 907–912. 10.1016/S0028-3932(96)00095-4 9204494

[B3] Abramovitch A. Cooperman A. (2015). The cognitive neuropsychology of obsessive-compulsive disorder: a critical review. *J. Obsessive Compuls. Relat. Disord.* 5 24–36. 10.1016/j.jocrd.2015.01.002

[B4] Altgassen M. Kliegel M. Rendell P. Henry J. D. Zöllig J. (2008). Prospective memory in schizophrenia: the impact of varying retrospective-memory load. *J. Clin. Exp. Neuropsychol.* 30 777–788. 10.1080/13803390701779552 18608664

[B5] Andreasen N. C. Nopoulos P. O’Leary D. S. Miller D. D. Wassink T. Flaum M. (1999). Defining the phenotype of schizophrenia: cognitive dysmetria and its neural mechanisms. *Biol. Psychiatry* 46 908–920. 10.1016/S0006-3223(99)00152-3 10509174

[B6] Andreasen N. C. Rezai K. Alliger R. Swayze V. W. II Flaum M. Kirchner P. (1992). Hypofrontality in neuroleptic-naive patients and in patients with chronic schizophrenia. *Arch. Gen. Psychiatry* 49 943–958. 10.1001/archpsyc.1992.01820120031006 1360199

[B7] American Psychiatric Association (2000). *Diagnostic and Statistical Manual of Mental Disorders (DSM–IV)*, 4th Edn. Washington, DC: American Psychiatric Association.

[B8] Baddeley A. D. (1986). *Working Memory.* Oxford: Clarendon Press.

[B9] Baddeley A. D. (2001). Is working memory still working? *Am. Psychol.* 56 851–864. 10.1037/0003-066X.56.11.851 11785152

[B10] Baddeley A. D. Hitch G. J. (1974). “Working memory,” in *The Psychology of Learning and Motivation* Vol. 8 ed. Bower G. (New York: Academic Press), 47–90.

[B11] Balázs J. Bitter I. Hideg K. Vitrai J. (1998). A M.I.N.I. és a M.I.N.I. Plussz kérdőív magyar nyelvű változatának kidolgozása (Development of the Hungarian version of the M.I.N.I. and M.I.N.I. Plus questionnaires). *Psychiatr. Hung.* 13 160–168.

[B12] Benoit R. G. Gilbert S. J. Frith C. D. Burgess P. W. (2012). Rostral prefrontal cortex and the focus of attention in prospective memory. *Cereb. Cortex* 22 1876–1886. 10.1093/cercor/bhr264 21976356 PMC3388891

[B13] Berman K. F. Weinberger D. R. (1991). “Functional localization in the brain in schizophrenia,” in *Review of Psychiatry* Vol. 10 eds Tasman A. Goldfinger S. (Washington, DC: American Psychiatric Press), 24–59.

[B14] Bisiacchi P. S. (1996). “The neuropsychological approach in the study of prospective memory,” in *Prospective Memory: Theory and Applications*, eds Brandimonte M. Einstein G. O. McDaniel M. A. (Mahwah, NJ: Lawrence Erlbaum Associates Inc.), 297–318.

[B15] Brunfaut E. Vanoverberghe V. D’Ydewalle G. (2000). Prospective remembering of Korsakoffs and alcoholics as a function of the prospective-memory and on-going tasks. *Neuropsychologia* 38 975–984. 10.1016/S0028-3932(00)00016-6 10775708

[B16] Burgess P. W. (2000). Strategy application disorder: the role of the frontal lobes in human multitasking. *Psychol. Res.* 63 279–288. 10.1007/s004269900006 11004881

[B17] Burgess P. W. Quayle A. Frith C. D. (2001). Brain regions involved in prospective memory as determined by positron emission tomography. *Neuropsychologia* 39 545–555. 10.1016/S0028-3932(00)00149-4 11257280

[B18] Burgess P. W. Scott S. K. Frith C. D. (2003). The role of the rostral frontal cortex (area 10) in prospective memory: a lateral versus medial dissociation. *Neuropsychologia* 41 906–918. 10.1016/S0028-3932(02)00327-5 12667527

[B19] Chan R. C. K. Wang Y. Ma Z. Hong X. Yuen Y. Yu X. (2008). Objective measures of prospective memory do not correlate with subjective complaints in schizophrenia. *Schizophr. Res.* 103 229–239. 10.1016/j.schres.2008.02.019 18420383

[B20] Cheung E. F. Lui S. S. Wang Y. Yang T. X. Shum D. H. Chan R. C. (2015). Time-based but not event-based prospective memory remains impaired one year after the onset of schizophrenia: a prospective study. *Schizophr. Res.* 169 147–152. 10.1016/j.schres.2015.09.015 26404040

[B21] Cohen J. D. O’Reilly R. C. (1996). “A preliminary theory of the interactions between prefrontal cortex and hippocampus that contribute to planning and prospective memory,” in *Prospective Memory: Theory and Applications*, eds Brandimonte M. Einstein G. O. McDaniel M. A. (New York, NY: Lawrence Erlbaum Associates Inc), 267–296.

[B22] Daum I. Mayes A. R. (2000). Memory and executive function impairments after frontal or posterior cortex lesions. *Behav. Neurol.* 12 161–173. 10.1155/2000/327304 11568428

[B23] Einstein G. O. McDaniel M. A. (1996). “Retrieval processes in prospective memory: theoretical approaches and some new empirical findings,” in *Prospective Memory: Theory and Applications*, eds Brandimonte M. Einstein G. O. McDaniel M. A. (New York, NY: Lawrence Erlbaum Associates Inc.), 115–141.

[B24] Ellis J. (1996). “Prospective memory or the realization of delayed intentions: a conceptual framework for research,” in *Prospective Memory: Theory and Applications*, eds Brandimonte M. Einstein G. O. McDaniel M. A. (New York, NY: Lawrence Erlbaum Associates Inc.), 1–22.

[B25] Elvevåg B. Maylor E. A. Gilbert A. L. (2003). Habitual prospective memory in schizophrenia. *BMC Psychiatry* 3:3 10.1186/1471-244X-3-3 PMC184442 12890293

[B26] Fortin S. Godbout L. Braun C. M. J. (2002). Strategic sequence planning and prospective memory impairments in frontally lesioned head trauma patients performing activities of daily living. *Brain Cogn.* 48 361–365. 12030468

[B27] Fortin S. Godbout L. Braun C. M. J. (2003). Cognitive structure of executive deficits in frontally lesioned head trauma patients performing activities of daily living. *Cortex* 39 273–291. 10.1016/S0010-9452(08)70109-6 12784889

[B28] Friston K. J. Frith C. D. (1995). Schizophrenia: a disconnection syndrome? *Clin. Neurosci.* 3 89–97. 7583624

[B29] Graf P. Uttl B. (2001). Prospective memory: a new focus for research. *Consc. Cogn.* 10 437–450. 10.1006/ccog.2001.0504 11790035

[B30] Green M. F. Harvey P. D. (2014). Cognition in schizophrenia: past, present, and future. *Schizophr. Res. Cogn.* 1:1 10.1016/j.scog.2014.02.001 PMC4171037 25254156

[B31] Guynn M. J. (2003). A two-process model of monitoring in eventbased prospective memory: activation/retrieval mode and checking. *Int. J. Psychol.* 38 245–256. 10.1080/00207590344000178

[B32] Guynn M. J. (2008). “Theory of monitoring in prospective memory: instantiating a retrieval mode and periodic target checking,” in *Prospective Memory: Cognitive, Neuroscience, Developmental, and Applied Perspectives*, eds Kliegel M. McDaniel M. A. Einstein G. O. (New York, NY: Lawrence Erlbaum Associates Inc.), 53–76.

[B33] Guynn M. J. McDaniel M. A. Einstein G. O. (2001). “Remembering to perform actions: a different type of memory? in *Memory for Action: A Distinct form of Episodic Memory?* eds Zimmer H. D. Cohen R. L. Guynn M. J. Engelkamp J. Kormi-Nouri R. Foley M. A. (New York, NY: Oxford University Press), 25–48.

[B34] Heinrichs R. W. Zakzanis K. K. (1998). Neurocognitive deficit in schizophrenia: a quantitative review of the evidence. *Neuropsychology* 12 426–445. 10.1037/0894-4105.12.3.426 9673998

[B35] Henry J. D. Rendell P. G. Kliegel M. Altgassen M. (2007). Prospective memory in schizophrenia: primary or secondary impairment? *Schizophr. Res.* 95 179–185. 10.1016/j.schres.2007.06.003 17630257

[B36] Kay S. R. Fiszbein A. Opfer L. A. (1987). The positive and negative syndrome scale (PANSS) for schizophrenia. *Schizophr. Bull.* 13 261–276. 10.1093/schbul/13.2.261 3616518

[B37] Kliegel M. Altgassen M. Hering A. Rose N. (2011). A process-model based approach to prospective memory impairment in Parkinson’s disease. *Neuropsychologia* 49 2166–2177. 10.1016/j.neuropsychologia.2011.01.024 21255592

[B38] Kliegel M. Eschen A. Thöne-Otto A. I. T. (2004a). Planning and realization of complex intentions in traumatic brain injury and normal aging. *Brain Cogn.* 56 43–54. 10.1016/j.bandc.2004.05.005 15380875

[B39] Kliegel M. Mackinlay R. J. Jäger T. (2008). “Prospective memory development: a lifespan approach,” in *Prospective Memory: Cognitive, Neuroscience, Developmental, and Applied Perspectives*, eds Kliegel M. McDaniel M. A. Einstein G. O. (Mahwah: Erlbaum), 187–216.

[B40] Kliegel M. Martin M. McDaniel M. A. Einstein G. O. (2001). Varying the importance of a prospective memory task: differential effects across time- and event-based prospective memory. *Memory* 9 1–11. 10.1080/09658210042000003 11315657

[B41] Kliegel M. Martin M. McDaniel M. A. Einstein G. O. (2002). Complex prospective memory and executive control of working memory: a process model. *Psychol. Beiträge* 44 303–318.

[B42] Kliegel M. Martin M. McDaniel M. A. Einstein G. O. (2004b). Importance effects on performance in event-based prospective memory tasks. *Memory* 12 553–561. 10.1080/09658210344000099 15615314

[B43] Kondel T. K. (2002). Prospective memory and executive function in schizophrenia. *Brain Cogn.* 48 405–410. 12030477

[B44] Kopp U. A. Thöne-Otto A. I. T. (2003). Disentangling executive functions and memory processes in event-based prospective remembering after brain damage: a neuropsychological study. *Int. J. Psychol.* 38 229–235. 10.1080/00207590344000150

[B45] Kuelz A. K. Hohagen F. Voderholzer U. (2004). Neuropsychological performance in obsessive-compulsive disorder: a critical review. *Biol. Psychol.* 65 185–236. 10.1016/j.biopsycho.2003.07.007 14757309

[B46] Lui S. S. Wang Y. Yang T. X. Liu A. C. Chui W. W. Yeung H. K. (2014). Problems in remembering to carry out future actions in first-episode schizophrenia: primary or secondary impairment? *J. Psychiatr. Res.* 61 141–149. 10.1016/j.jpsychires.2014.11.007 25479767

[B47] Lui S. Y. Wang Y. Liu A. Y. Chui W. H. Gong Q. Shum D. (2011). Prospective memory in patients with first-onset schizophrenia and their nonpsychotic siblings. *Neuropsychologia* 49 2217–2224. 10.1016/j.neuropsychologia.2011.04.002 21507327

[B48] Manoach D. S. (2003). Prefrontal cortex dysfunction during working memory performance in schizophrenia: reconciling discrepant findings. *Schizophr. Res.* 60 285–298. 10.1016/S0920-9964(02)00294-3 12591590

[B49] Marsh R. L. Hicks J. L. Cook G. I. (2005). On the relationship between effort toward an ongoing task and cue detection in event-based prospective memory. *J. Exp. Psychol. Learn. Mem. Cogn.* 31 68–75. 10.1037/0278-7393.31.1.68 15641905

[B50] Matza L. S. Buchanan R. Purdon S. Brewster-Jordan J. Zhao Y. Revicki D. A. (2006). Measuring changes in functional status among patients with schizophrenia: the link with cognitive impairment. *Schizophr. Bull.* 32 666–678. 10.1093/schbul/sbl004 16829550 PMC2632260

[B51] Maujean A. Shum D. McQueen R. (2003). Effect of cognitive demand on prospective memory in individuals with traumatic brain injury. *Brain Impair.* 4 135–145. 10.1375/brim.4.2.135.27024

[B52] McDaniel M. A. Einstein G. O. (2000). Strategic and automatic processes in prospective memory retrieval: a multiprocess framework. *Appl. Cogn. Psychol.* 14 S127–S144. 10.1002/acp.775

[B53] McDaniel M. A. Glisky E. L. Rubin S. R. Guynn M. J. Routhieaux B. C. (1999). Prospective memory: a neuropsychological study. *Neuropsychology* 13 103–110. 10.1037/0894-4105.13.1.103 10067781

[B54] McDaniel M. A. Guynn M. J. Einstein G. O. Breneiser J. (2004). Cue-focused and reflexive-associative processes in prospective memory retrieval. *J. Exp. Psychol. Learn. Mem. Cogn.* 30 605–614. 10.1037/0278-7393.30.3.605 15099129

[B55] Meyer-Lindenberg A. Miletich R. S. Kohn P. D. Esposito G. Carson R. E. Quarantelli M. (2002). Reduced prefrontal activity predicts exaggerated striatal dopaminergic function in schizophrenia. *Nat. Neurosci.* 5 267–271. 10.1038/nn804 11865311

[B56] Miyake A. Friedman N. P. Emerson M. J. Witzki A. H. Howerter A. Wager T. D. (2000). The unity and diversity of executive functions and their contributions to complex “Frontal Lobe” tasks: a latent variable analysis. *Cogn. Psychol.* 41 49–100. 10.1006/cogp.1999.0734 10945922

[B57] Nieuwenstein M. R. Aleman A. de Haan E. H. (2001). Relationship between symptom dimensions and neurocognitive functioning in schizophrenia: a meta-analysis of WCST and CPT studies. *J. Psychiatr. Res.* 35 119–125. 10.1016/S0022-3956(01)00014-0 11377441

[B58] Okuda J. Fujii T. Yamadori A. Kawashima R. Tsukiura T. Fukatsu R. (1998). Participation of the prefrontal cortices in prospective memory: evidence from a PET study in humans. *Neurosci. Lett.* 253 127–130. 10.1016/S0304-3940(98)00628-4 9774166

[B59] Olley A. Malhi G. Sachdev P. (2007). Memory and executive functioning in obsessive-compulsive disorder: a selective review. *J. Affect. Disord.* 104 15–23. 10.1016/j.jad.2007.02.023 17442402

[B60] Ordemann G. J. Opper J. Davalos D. (2014). Prospective memory in schizophrenia: a review. *Schizophr. Res.* 155 77–89. 10.1016/j.schres.2014.03.008 24698096

[B61] Perlstein W. M. Carter C. S. Noll D. C. Cohen J. D. (2001). Relation of prefrontal cortex dysfunction to working memory and symptoms in schizophrenia. *Am. J. Psychiatry* 158 1105–1113. 10.1176/appi.ajp.158.7.1105 11431233

[B62] Racsmány M. Conway M. A. Garab E. A. Cimmer C. Janka Z. Kurimay T. (2008). Disrupted memory inhibition in schizophrenia. *Schizophr. Res.* 101 218–224. 10.1016/j.schres.2008.01.002 18258417

[B63] Racsmány M. Demeter G. Csigó K. Harsányi A. Németh A. (2011). An experimental study of prospective memory in obsessive-compulsive disorder. *J. Clin. Exp. Neuropsychol.* 33 85–91. 10.1080/13803395.2010.493147 20614363

[B64] Raffard S. Bayard S. (2012). Understanding the executive functioning heterogeneity in schizophrenia. *Brain Cogn.* 79 60–69. 10.1016/j.bandc.2012.01.008 22342280

[B65] Ranganath C. Minzenberg M. J. Ragland J. D. (2008). The cognitive neuroscience of memory function and dysfunction in schizophrenia. *Biol. Psychiatry* 64 18–25. 10.1016/j.biopsych.2008.04.011 18495087 PMC2474810

[B66] Sheehan D. V. Lecrubier Y. Sheehan K. H. Amorim P. Janavs J. Weiller E. (1998). The Mini-International Neuropsychiatric Interview (M.I.N.I.): the development and validation of a structured diagnostic psychiatric interview for DSM-IV and ICD-10. *J. Clin. Psychiatry* 59 22–33. 9881538

[B67] Shum D. Leung J. P. Ungvari G. S. Tang W. K. (2001). Schizophrenia and prospective memory: a new direction for clinical practice and research? *Hong Kong J. Psychiatry* 11 23–26.

[B68] Shum D. Ungvari G. S. Tang W. Leung J. (2004). Performance of schizophrenia patients on time-, event-, and activity-based prospective memory tasks. *Schizophr. Bull.* 30 693–701. 10.1093/oxfordjournals.schbul.a007123 15954184

[B69] Simons J. S. Schölvinck M. L. Gilbert S. J. Frith C. D. Burgess P. W. (2006). Differential components of prospective memory? *Neuropsychology* 44 1388–1397. 10.1016/j.neuropsychologia.2006.01.005 16513147

[B70] Smith R. E. (2003). The cost of remembering to remember in event-based prospective memory: investigating the capacity demands of delayed intention performance. *J. Exp. Psychol. Learn. Mem. Cogn.* 29 347–361. 10.1037/0278-7393.29.3.347 12776746

[B71] Smith R. E. Bayen U. J. (2004). A multinomial model of event-based prospective memory. *J. Exp. Psychol. Learn. Mem. Cogn.* 30 756–777. 10.1037/0278-7393.30.4.756 15238021

[B72] Snitz B. E. Macdonald A. W. III Carter C. S. (2006). Cognitive deficits in unaffected first-degree relatives of schizophrenia patients: a meta-analytic review of putative endophenotypes. *Schizophr. Bull.* 32 179–194. 10.1093/schbul/sbi048 16166612 PMC2632195

[B73] Stephan K. E. Baldeweg T. Friston K. J. (2006). Synaptic plasticity and dysconnection in schizophrenia. *Biol. Psychiatry* 59 929–939. 10.1016/j.biopsych.2005.10.005 16427028

[B74] Twamley E. W. Woods S. Zurhellen C. H. Vertinski M. Narvaez J. M. Mausbach B. T. (2008). Prospective memory and its correlates and predictors in schizophrenia: an extensions of previous findings. *Arch. Clin. Neuropsychol.* 23 613–622. 10.1016/j.acn.2008.06.005 18635339

[B75] Ungvari G. S. Xiang Y. Tang W. Shum D. (2008). Prospective memory and its correlates and predictors in schizophrenia: an extension of previous findings. *Arch. Clin. Neuropsychol.* 23 613–622. 10.1016/j.acn.2008.06.005 18635339

[B76] Velligan D. I. Bow-Thomas C. C. (1999). Executive function in schizophrenia. *Semin. Clin. Neuropsychiatry* 4 24–33. 10229790 10.1053/SCNP00400024

[B77] Volz H. P. Gaser C. Häger F. Rzanny R. Mentzel H. J. Kreitschmann-Andermahr I. (1997). Brain activation during cognitive stimulation with the Wisconsin Card Sorting Test – a functional MRI study on healthy volunteers and schizophrenics. *Psychiatry Res. Neuroimag.* 75 145–157. 10.1016/S0925-4927(97)00053-X 9437772

[B78] Wang Y. Chan R. C. K. Hong X. Ma Z. Yang T. Guo L. (2008). Prospective memory in schizophrenia: further clarification of nature of impairment. *Schizophr. Res.* 105 114–124. 10.1016/j.schres.2008.07.002 18707848

[B79] Wang Y. Cui J. Chan R. C. Deng Y. Shi H. Hong X. (2009). Meta-analysis of prospective memory in schizophrenia: nature, extent, and correlates. *Schizophr. Res.* 114 64–70. 10.1016/j.schres.2009.07.009 19713081

[B80] Woods S. P. Twamley E. W. Dawson M. S. Narvaez J. M. Jeste D. V. (2007). Deficits in cue detection and intention retrieval underlie prospective memory impairment in schizophrenia. *Schizophr. Res.* 90 344–350. 10.1016/j.schres.2006.11.005 17175138 PMC1851918

[B81] Zhou F. Xiang Y. Wang C. Dickerson F. Au R. C. Zhou J. (2012). Characteristics and clinical correlates of prospective memory performance in first-episode schizophrenia. *Schizophr. Res.* 135 34–39. 10.1016/j.schres.2011.12.001 22222379

[B82] Zhuo K. Lu Y. Yang Z. Fan X. Song Z. Liao L. (2013). Prospective memory performance in patients with drug-naïve, first-episode psychosis. *Schizophr. Res.* 143 285–290. 10.1016/j.schres.2012.12.002 23267733

[B83] Zöllig J. West R. Martin M. Altgassen M. Lemke U. Kliegel M. (2007). Neural correlates of prospective memory across the lifespan. *Neuropsychologia* 45 3299–3314. 10.1016/j.neuropsychologia.2007.06.010 17675111

